# Supplementary material for: Conserved Role of Heterotrimeric G Proteins in Plant Defense and Cell Death Progression
Source: Genes (Basel). 2024 Jan 18;15(1):115. doi: 10.3390/genes15010115 (PMC10815853; doi:10.3390/genes15010115)
Supplement: Supplementary file 1 [file genes-15-00115-s001.zip › genes-2823528-supplementary.pdf]

Supplementary data

Table S1. Primers used for amplification and cloning of G-protein genes.

Primer Name	Sequence
AGB1 Forward (XbaI)	TCTAGAATGGTGAGCGGCTGGCGGCTGTTCAAGAAG ATTAGCATGTCTGTCTCCGAG
AGB1 Reverse (KpnI)	TGGTACCTCAAATCACTCTCCTGTGTC
AGG1 Forward (XbaI)	TCTAGAATGGTGAGCGGCTGGCGGCTGTTCAAGAAG ATTAGCATGGGAGAGGAAACT
AGG1 Reverse (KpnI)	TGGTACCTCAAAGTATTAAGCATCTGCAGCCT
SlGB1 Forward (XbaI)	TTTCTAGACCATGGCAGTTGCGGAGCTGAAAGA
SlGB1 Reverse (KpnI)	AAGGTACCTCAGATCACACTTCTGTGTCCT
SlGGA1 Forward (XbaI)	TTTCTAGACCATGGAATCAGAGAGCTCAGAACA
SlGGA1 Reverse (KpnI)	AAGGTACCTCACAATATCCAGCATCTGCAAC
XLG2 Forward (XbaI)	GCTCTAGAATGGCTGCAGTTATAAGAAAGTTATTAC
XLG2 Reverse (KpnI)	GGTACCTCAAGAGGACGAGCTG

Table S2. Primers used for RT-qPCR.

SAND-F	GTTGGGTCACACCAGATTTTG
SAND-R	GCTCCTTGCAAGAACACTTCA
RT-PR1-F	AAGAGGCAACTGCAGACTCA
RT-PR1-R	TCTCGCTAACCCACATGTTC
RT-PR2-F	GATTCTTCTCAGCCTTGTAATAGCTTC
RT-PR2-R	GATGAGCTCGATGTCAGAGCCAC
RT-PR3-F	ACGGAAGAGGACCAATGCAA
RT-PR3-R	GAGCAGTCATCCAGAACCAAATC
RT-PR4-F	ATCATACACAGTGGCTACGGTG
RT-PR4-R	CTTCCCCAACAATTGCTCTGAC
RT-PR5-F	TCGGCGATGGAGGATTTGAA
RT-PR5-R	AGCCAGAGTGACGGGAGGAAC

Figure S1. Complete maps of expression vectors used in this study

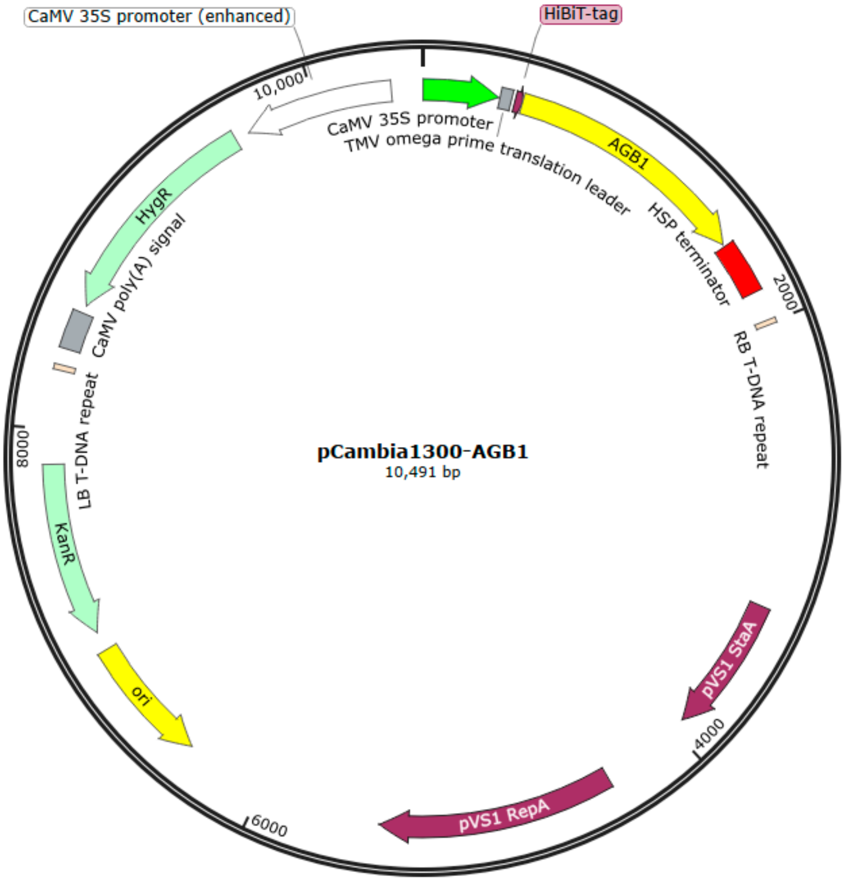
CaMV 35S promoter (enhanced)
HiBiT-tag
10,000
CaMV 35S promoter
TMV omega prime translation leader
AGB1
HSP terminator
2000
RB T-DNA repeat
HygR
CaMV poly(A) signal
8000
LB T-DNA repeat
KanR
ori
pVS1 StaA
4000
pVS1 RepA
6000
pCambia1300-AGB1
10,491 bp

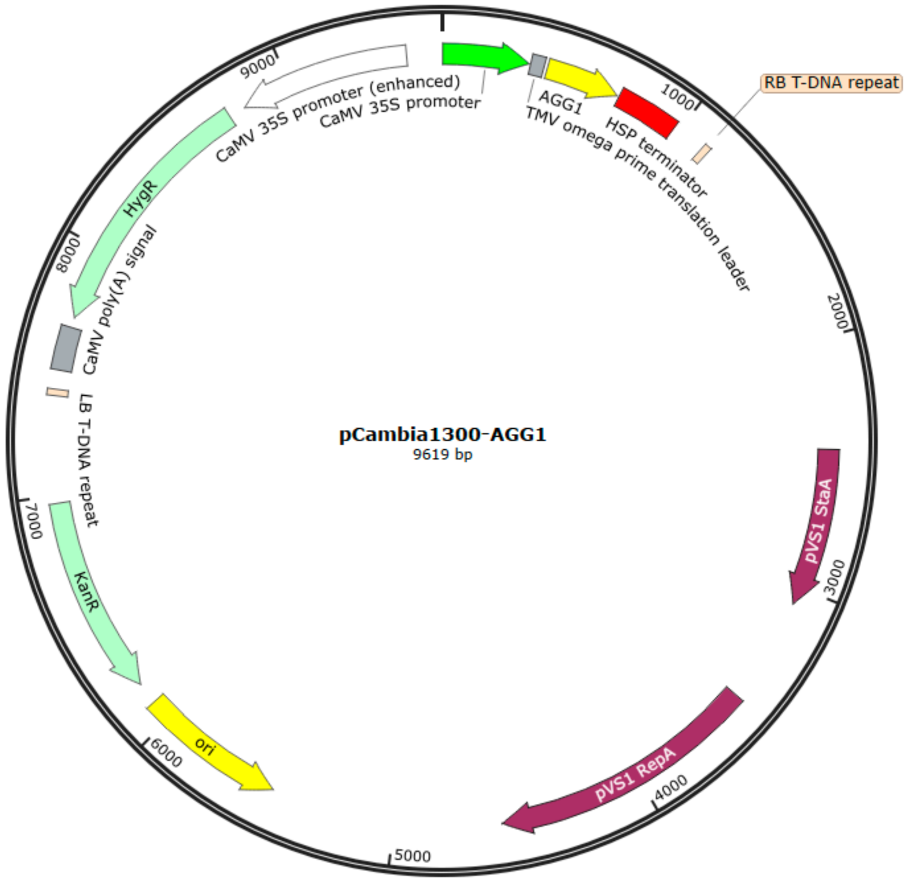
RB T-DNA repeat
9000
CaMV 35S promoter (enhanced)
CaMV 35S promoter
AGG1
TMV omega prime translation leader
HSP terminator
1000
HygR
8000
CaMV poly(A) signal
LB T-DNA repeat
2000
pCambia1300-AGG1
9619 bp
7000
KanR
pVS1 StaA
3000
ori
6000
pVS1 RepA
4000
5000

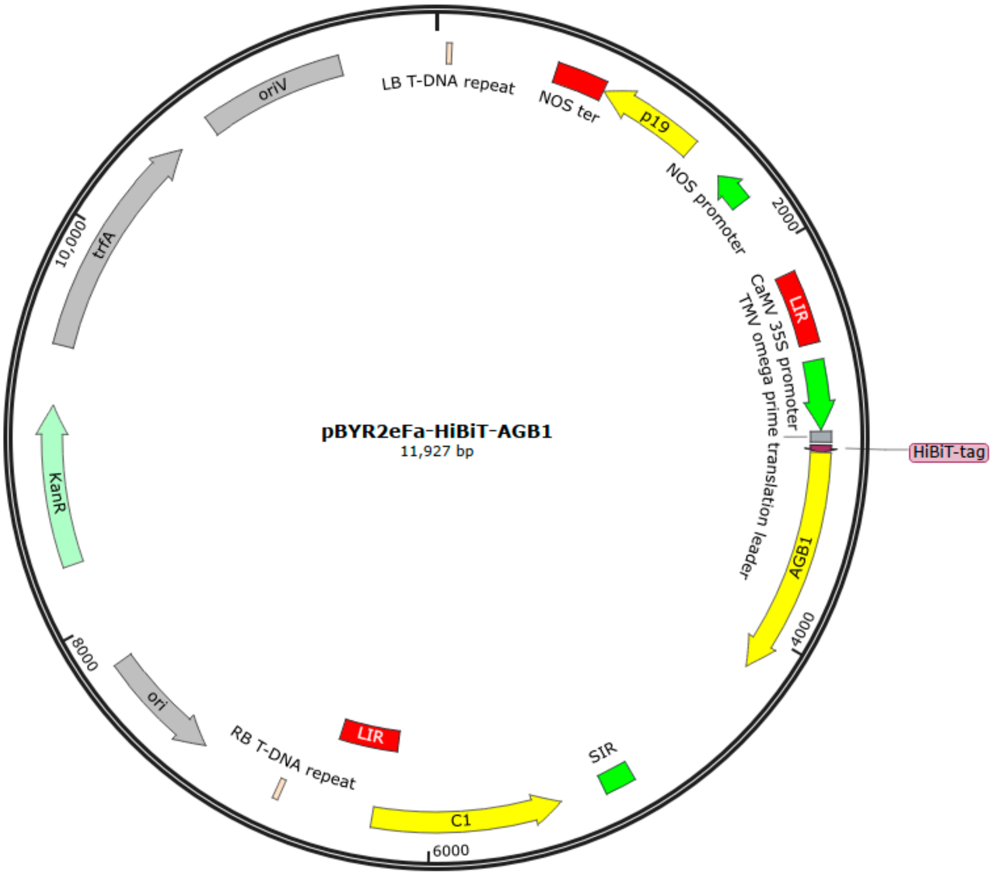
pBYR2eFa-HiBiT-AGB1
11,927 bp
LB T-DNA repeat
NOS ter
p19
NOS promoter
2000
LIR
CaMV 35S promoter
TMV omega prime translation leader
HiBiT-tag
AGB1
4000
SIR
C1
6000
LIR
RB T-DNA repeat
ori
8000
KanR
trfA
10,000
oriV

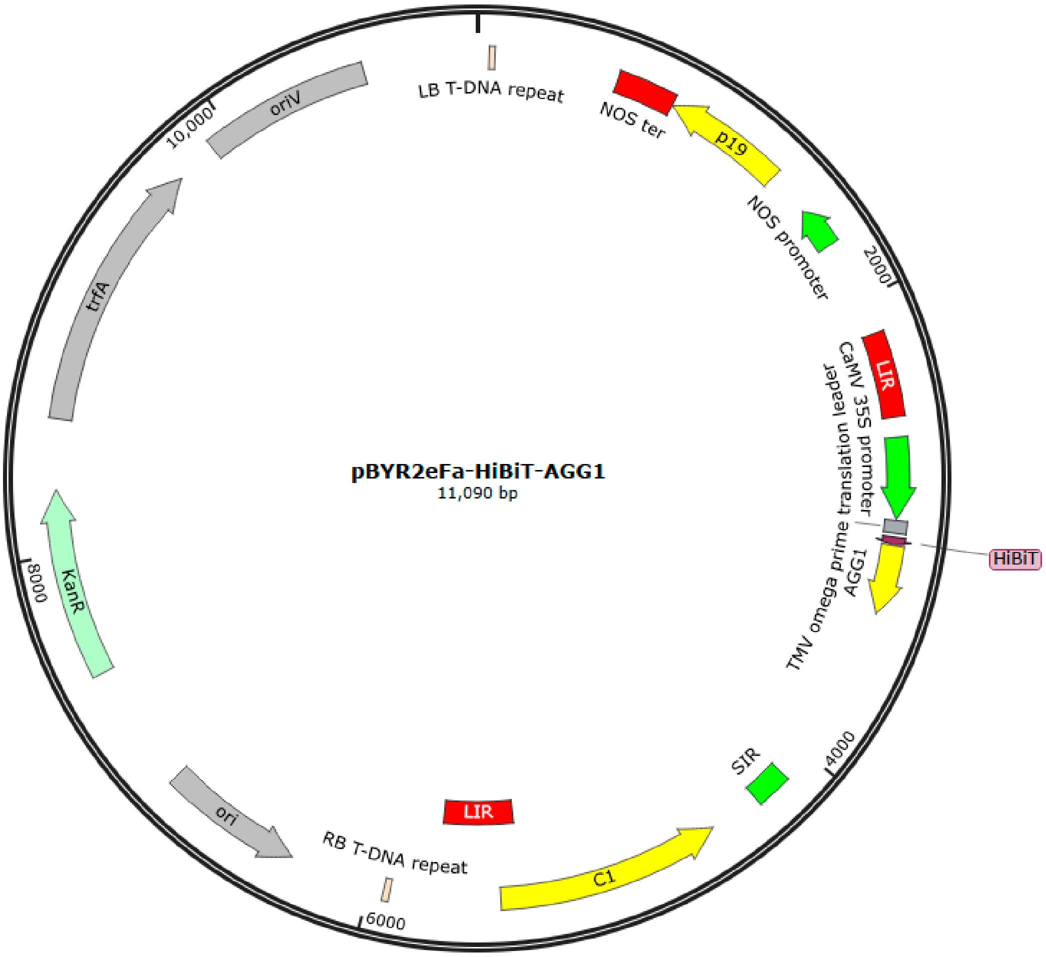
pBYR2eFa-HiBiT-AGG1
11,090 bp
LB T-DNA repeat
NOS ter
p19
NOS promoter
2000
LIR
CaMV 35S promoter
TMV omega prime translation leader
HiBiT
AGG1
4000
SIR
C1
6000
LIR
RB T-DNA repeat
ori
8000
KanR
trfA
10,000
oriV

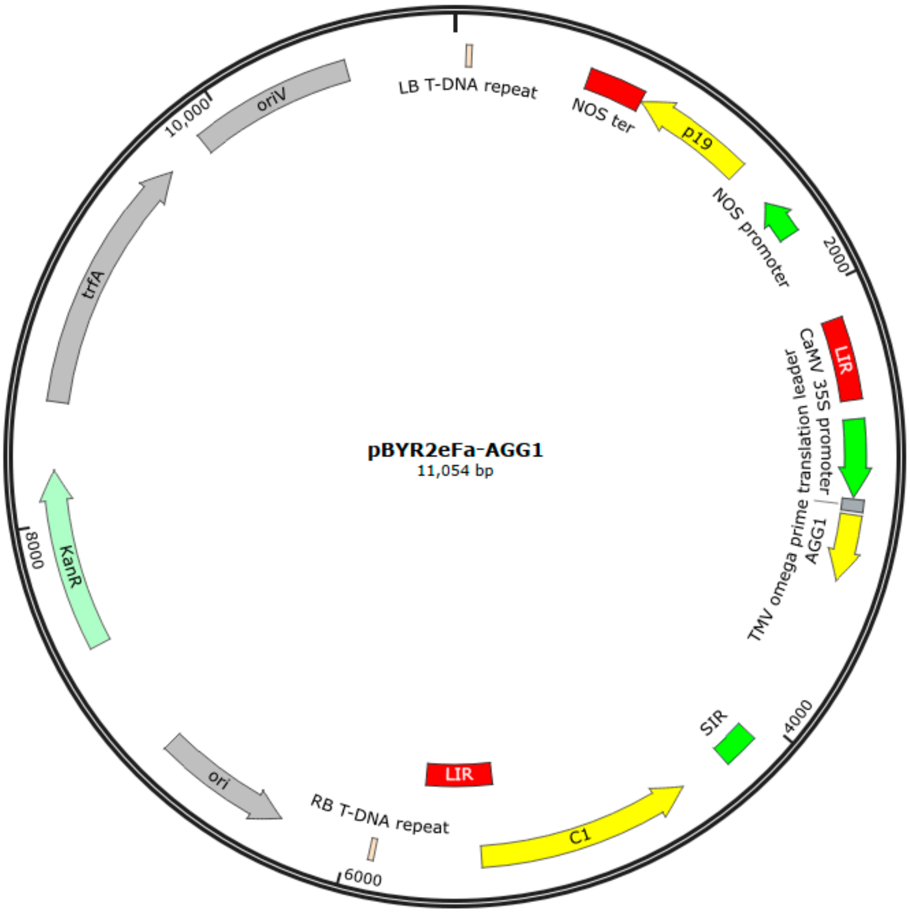
pBYR2eFa-AGG1
11,054 bp
LB T-DNA repeat
NOS ter
p19
NOS promoter
2000
LIR
CaMV 35S promoter
TMV omega prime translation leader
AGG1
4000
SIR
C1
LIR
RB T-DNA repeat
6000
ori
KanR
8000
trfA
10,000
oriV

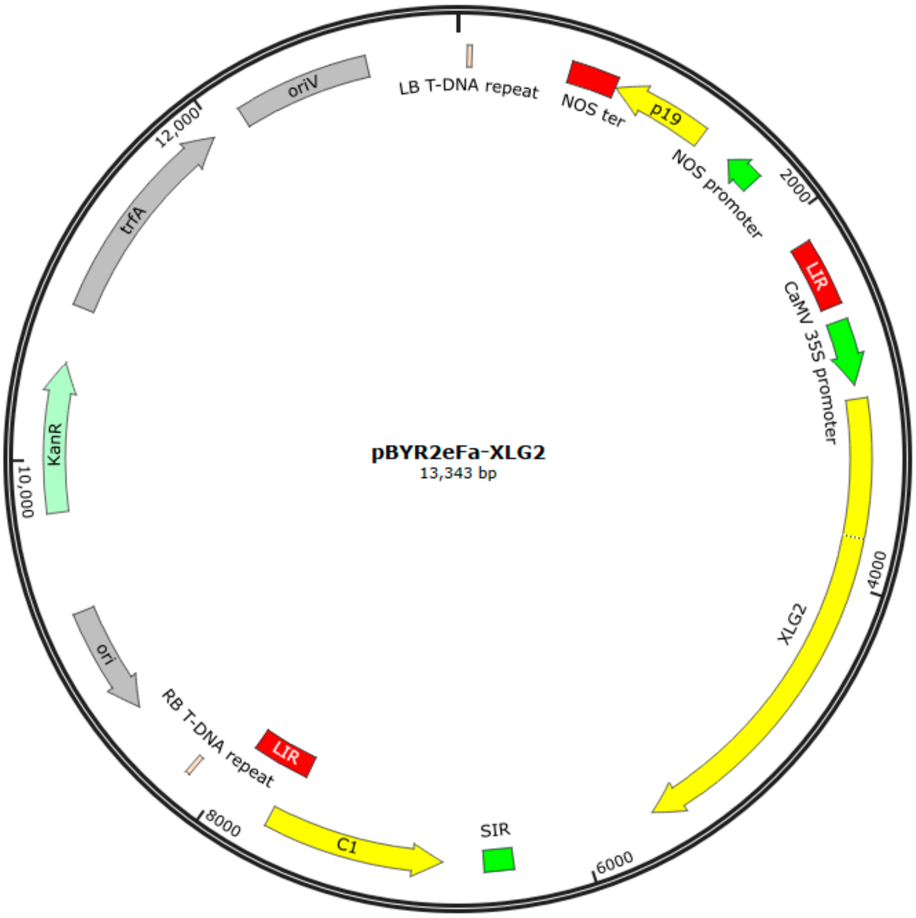
pBYR2eFa-XLG2
13,343 bp
LB T-DNA repeat
NOS ter
p19
NOS promoter
2000
LIR
CaMV 35S promoter
4000
XLG2
6000
SIR
C1
LIR
RB T-DNA repeat
8000
ori
KanR
10,000
trfA
12,000
oriV

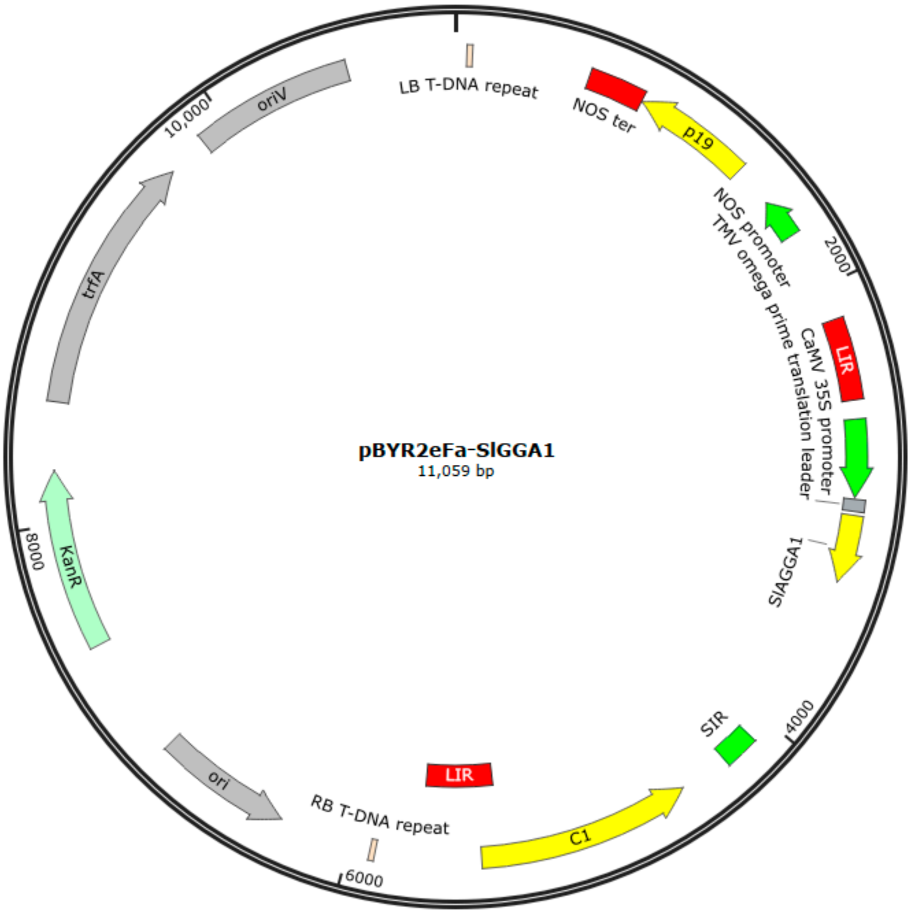
pBYR2eFa-SlGGA1
11,059 bp
LB T-DNA repeat
NOS ter
p19
NOS promoter
TMV omega prime translation leader
CaMV 35S promoter
LIR
SlAGGA1
SIR
C1
LIR
RB T-DNA repeat
ori
KanR
trfA
oriV
2000
4000
6000
8000
10,000

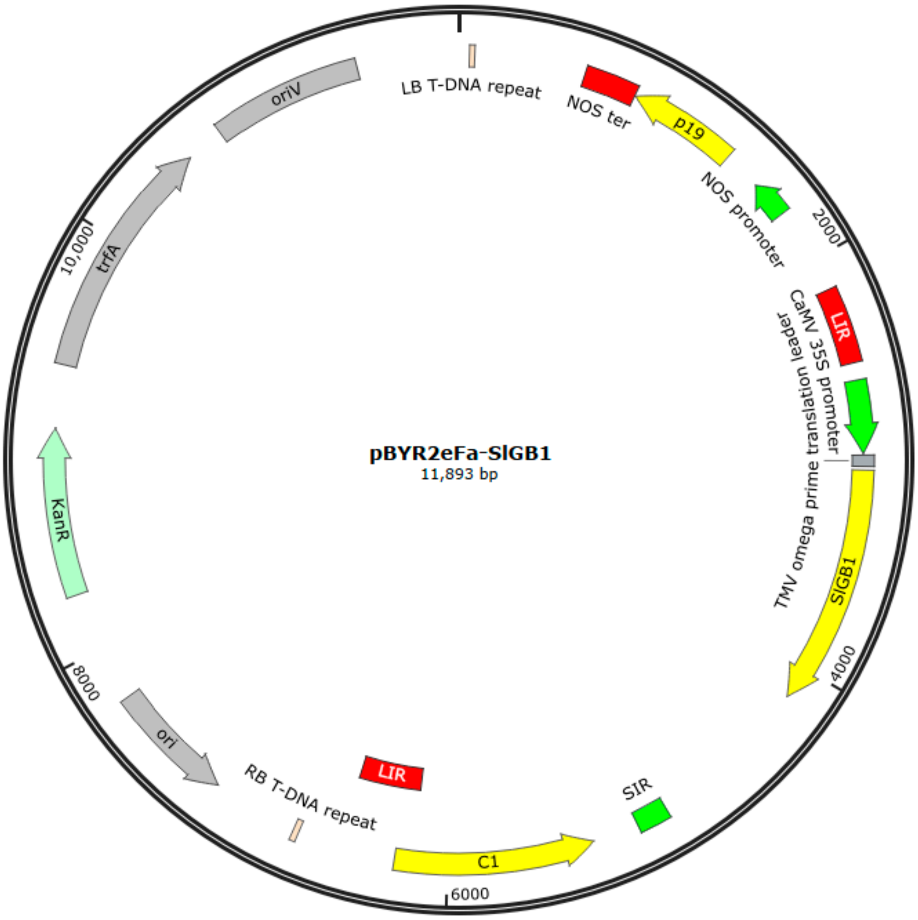
pBYR2eFa-SlGB1
11,893 bp
LB T-DNA repeat
NOS ter
p19
NOS promoter
LIR
CaMV 35S promoter
TMV omega prime translation leader
SlGB1
SIR
C1
LIR
RB T-DNA repeat
ori
KanR
trfA
oriV
2000
4000
6000
8000
10,000
